# Torsion ovarienne en post-partum: à propos d’un cas

**DOI:** 10.11604/pamj.2019.33.240.19309

**Published:** 2019-07-19

**Authors:** Houda Chattri, Meryem Kouara, Khadija Chetouani

**Affiliations:** 1 Service de Radiologie, Centre Hospitalier Provincial de Guercif, Maroc; 2 Service de Gynéco-obstétrique, Centre Hospitalier Provincial de Guercif, Maroc; 3 Service de Réanimation, Centre Hospitalier Provincial de Guercif, Maroc

**Keywords:** Algie pelvienne, torsion d´annexe, post-partum, Pelvic pain, adnexal torsion, post-partum

## Abstract

La torsion d'ovaire est une étiologie rare de douleurs pelviennes en postpartum. L'absence de signes cliniques et radiologiques spécifiques rend la suspicion et le diagnostic préopératoire difficiles. Le retard de prise en charge peut mettre en jeu la viabilité de l'ovaire. Nous présentons le cas d'une patiente de 24 ans à J5 du postpartum qui s'est présentée aux urgences pour des douleurs pelviennes aiguës, chez qui le diagnostic de torsion ovarienne sur masse kystique a été fait sur la base des signes échographiques, permettant à la patiente de bénéficier d'une kystectomie et d'un traitement conservateur.

## Abstract (en)

Ovarian torsion is a rare cause of pelvic pain during the postpartum period. The absence of specific clinical and radiological signs makes suspicion and preoperative diagnosis difficult. Delayed management can compromise ovarian viability. We report the case of a 24-year patient presenting to the Emergency Department five days after delivery with severe pelvic pain. The diagnosis of ovarian torsion associated with cystic mass was made on the basis of ultrasound images. The patients underwent cystectomy and a conservative treatment.

## Introduction

La torsion d'annexe est une étiologie rare de douleurs pelviennes aiguës qui peut se voir à n'importe quel âge. Sa prévalence est de 2,5 -7,4% [1, 2] de toutes les urgences gynécologiques. Le diagnostic de la torsion demeure un challenge nécessitant une bonne connaissance des facteurs de haut risque et de la présentation clinique. La prédominance des causes plus classiques en période du post-partum (thrombose de la veine ovarienne, infections urinaires ou gynécologiques) et les tableaux atypiques de présentation rendent le diagnostic encore plus difficile, ce qui peut retarder la prise en charge et mettre en jeu le pronostic fonctionnel voire vital de la patiente. Nous présentons le cas d'une torsion d'annexe en post-partum chez une patiente de 24 ans dont le diagnostic a été radiologique.

## Patient et observation

**Figure 1:**
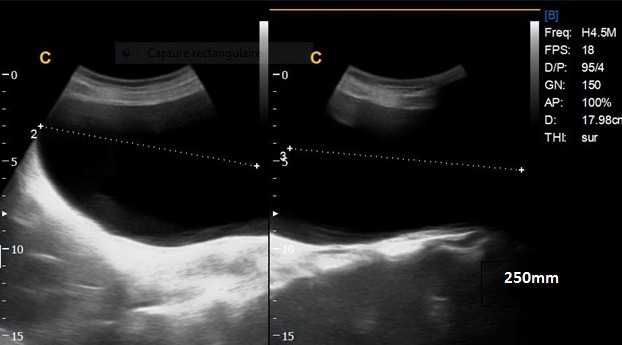
Masse kystique uniloculaire simple

**Figure 2:**
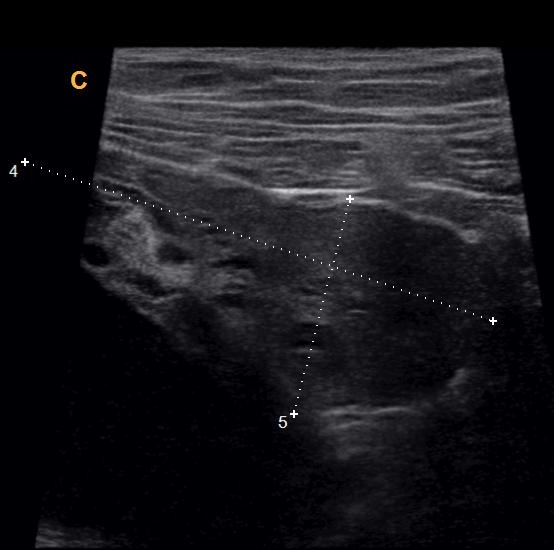
Ovaire droit hypertrophique et signe de l’éperon avec le kyste

**Figure 3:**
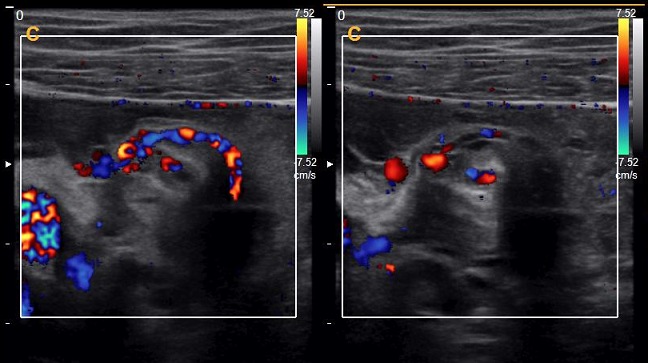
Image doppler de deux tours de spires du pédicule ovarien droit

**Figure 4:**
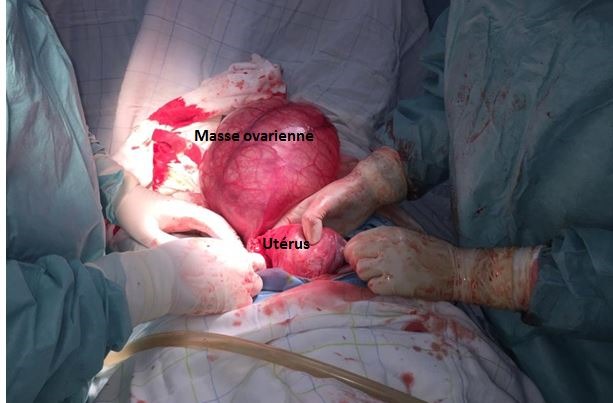
Image peropératoire montrant la masse ovarienne droite

**Figure 5:**
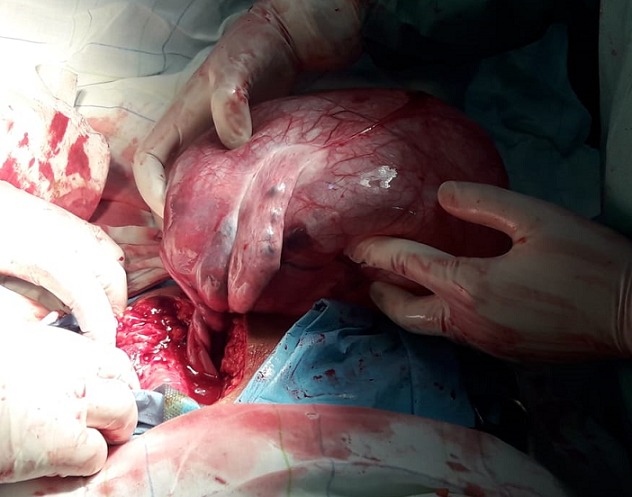
Image per-opératoire des tours de spires du pédicule ovarien droit

**Figure 6:**
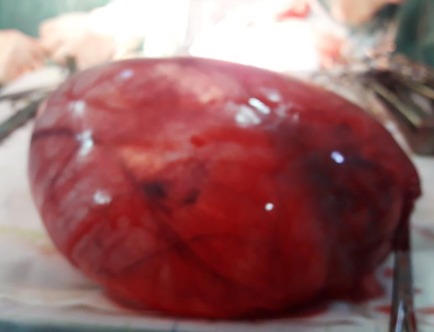
Image per-opératoire de la masse kystique après kystectomie

Une patiente de 24 ans, G1P1, s'est présentée au service des urgences pour des douleurs pelviennes aiguës survenues à J5 du post-partum d'un accouchement par voie basse. La symptomatologie est d'installation brutale évoluant depuis 4 heures. Elle est faite de douleurs aiguës du flanc droit, de nausées, sueurs froides et d'inconfort. Ses antécédents médicaux et chirurgicaux étaient sans particularités. L'examen clinique réalisé à l'admission trouve une patiente consciente, apyrétique, algique (EVA à 8), stable sur le plan hémodynamique. Une sensibilité abdominale est retrouvée à la palpation du flanc droit. Le bilan biologique initial trouve une anémie hypochrome microcytaire avec une Hb à 10 sans hyperleucytose associée. Les bandelettes urinaires étaient négatives. Une colique néphrétique a été suspectée et la patiente a été adressée au service de radiologie pour exploration. L'échographie réalisée par voie sus-pubienne retrouve une masse latéro-utérine droite de 250x50mm, arrivant à l'épigastre, bien limitée, d'échostructure liquidienne, uniloculaire, à paroi fine et à contenu anéchogène, sans portion charnue ou calcification visible (Figure 1). Un signe de l'éperon était retrouvé avec l'ovaire droit qui était hypertrophique mesurant 47x21mm (Figure 2). L'exploration par une sonde de haute fréquence retrouve en latéro-ovarien droit une image de deux tours de spires (Figure 3). La vascularisation en doppler couleur de l'ovaire était conservée. Une lame d'épanchement du CDS de Douglas était associée. L'utérus était globuleux. Il n'y avait pas de masse ovarienne gauche. L'examen abdomino-pelvien systématique ne trouvait pas d'anomalie échographique digestive ou urinaire observée. Le diagnostic d'une torsion ovarienne sur kyste ovarien simple a été retenu. La patiente a été conduite en urgence au bloc opératoire pour laparotomie. L'exploration chirurgicale retrouve la volumineuse masse ovarienne kystique droite (Figure 4). L'annexe droite était tordue de deux tours de spires (Figure 5). Le geste chirurgical a consisté en une détorsion de l'annexe et une kystectomie (Figure 6). En l'absence de signe ischémique ou hémorragique l'ovaire droit a été conservé. La douleur pelvienne s'est amendée en post-chirurgie. Le diagnostic anatomopathologique était en faveur d'un cystadénome séreux pesant 1832g. Les suites post-opératoires étaient sans particularités.

## Discussion

La torsion d'annexe est une urgence gynécologique qui demeure rare. Elle est définie comme une torsion partielle ou complète de l'ovaire et d'une portion tubaire autour de son pédicule vasculaire [3]. C'est une situation à faible prévalence qui peut se voir à n'importe quel âge avec un pic entre 20-30 ans. Le diagnostic est suspecté devant la présence d'un faisceau d'arguments combinant les antécédents de la patiente, les signes cliniques et les résultats échographiques. Huchon *et al*. [4] ont publié un score prédictif pour le diagnostic pré-opératoire de torsion, en identifiant cinq critères indépendamment associés à une torsion d'annexe, incluant: la douleur unilatérale, la durée de >8h, les vomissements, l'absence de leucorrhées ou de métrorragies et la présence d'un kyste de plus de 5 cm à l'échographie. Ce score permet l'identification de deux groupes de faible et haut risque. Les résultats de cette étude rétrospective sont intéressants mais nécessitent une confirmation prospective à plus grande échelle pour être retenus. L'absence de signes cliniques pathognomoniques rend la suspicion clinique difficile. En période du post-partum, cette difficulté est plus grande vue les complications plus classiques pouvant induire un retard diagnostique. Chez notre patiente, le diagnostic suspecté initialement était celui d'une colique néphrétique. Le recours à l'imagerie pour l'exploration des douleurs pelviennes aigues est fréquent. L'échographie est l'examen de première intention. L'apport de celle-ci pour le diagnostic positif de la torsion ovarienne reste controversé. La diversité des signes échographiques doivent être corrélés au tableau clinique de la patiente. L'intérêt de l'échographie est la recherche de facteurs favorisant une torsion d'annexe (hypertrophie ovarienne, masse), la recherche de complications (épanchement, pelvipéritonite) et le diagnostic différentiel des autres étiologies de douleurs pelviennes.

L'aspect typique de la torsion se manifeste par un élargissement ovarien unilatéral avec œdème et disposition périphérique des follicules [5]. La présence d'aspect en tours de spire a été démontrée comme un signe augmentant la sensibilité de l'examen [6]. L'absence de flux vasculaire annexiel au doppler est associée à 100% des cas de torsion [7]. Cependant, ce signe présente une faible valeur prédictive négative puisque l'absence totale de flux n'est visible que dans la phase tardive. Dans une étude 60% des torsions confirmées en chirurgie avaient un flux persistant [8]. Dans notre cas, le diagnostic a été posé en échographie après la mise en évidence de la volumineuse masse kystique et la visualisation des tours de spires du pédicule vasculaire. La rapidité du diagnostic a permis une prise en charge avant l'installation de l'ischémie ovarienne. Le recours au scanner pour l'exploration des algies pelviennes aux urgences est fréquent lorsqu'une cause urinaire ou digestive est suspectée. Sa spécificité et sa sensibilité dans le diagnostic des torsions ovariennes sont faibles. Hiller *et al.* ont montré que seulement 34% des torsions ovariennes confirmées chirurgicalement avaient un diagnostic préopératoire correct basé sur les résultats de la tomodensitométrie (TDM) [9, 10]. L'Imagerie par résonnance magnétique (IRM) est un examen non invasif performant dans le diagnostic des algies pelviennes et apporte un complément d'information à l'échographie. C'est un examen dont l'innocuité a été prouvée chez la femme enceinte. Le recours à cette technique en urgence reste limité par son cout important et sa faible disponibilité. Les masses ovariennes bénignes sont les causes les plus fréquentes des torsions d'annexe. La découverte d'une masse ovarienne durant la grossesse est comprise entre 0,3 et 5,4% [9, 10]. Les tumeurs organiques bénignes les plus fréquentes durant la grossesse sont les kystes dermoïdes et les cystadénomes [11]. Elles sont associées à un risque de torsion de 8% en post partum [11].

Vue le risque non négligeable de fausse couche en post-opératoire (2,8%) l'expectative est recommandée pour les tumeurs présumées bénignes de moins de 6 cm stables au cours de la grossesse. En cas de masse symptomatique ou compliquée, la chirurgie est indiquée quel que soit le terme [12]. En postpartum, le déplacement des annexes secondairement à l'involution physiologique de l'utérus et le relâchement des tissus de soutien augmentent la mobilité de la masse et le risque de torsion annexielle. Ce risque est estimé à 8,8% dans les trois semaines suivant un accouchement par voie basse [13]. Un suivi particulier et une chirurgie programmée devraient être de mise chez les parturientes porteuses de masses ovariennes présumées bénignes. Chez notre patiente, il s'agissait d'un cystadénome ovarien droit découvert en post-partum compliqué d'une torsion au 5^ème^ jour. La torsion d'annexe est une urgence chirurgicale. Le recours à la chirurgie est la règle devant la suspicion d'une torsion ovarienne afin de confirmer le diagnostic et d'éviter les dommages ovariens. L'exploration se fait le plus souvent par laparoscopie. La laparotomie est indiquée dans le cas d'une masse ovarienne de plus de 75 mm [14]. Le traitement radical (annexectomie) ou conservateur se fait en fonction de l'aspect de l'annexe après 10min après la détorsion [15]. Pour certains auteurs, le traitement conservateur est justifié malgré une vitalité douteuse de l'annexe [15]. Dans notre cas, le diagnostic radiologique de certitude en préopératoire de la patiente a permis d'instaurer une prise en charge. La laparotomie a été choisie devant le volume important de la masse, et l'absence d'ischémie de l'annexe a permis de réaliser une kystectomie et un traitement conservateur de l'ovaire.

## Conclusion

La torsion de l'ovaire est une urgence diagnostique et thérapeutique. Sa prévalence en postpartum est extrêmement rare. Sa suspicion clinique et son diagnostic préopératoire sont difficiles en raison de l'absence de signes spécifiques. La connaissance des facteurs de risque et des signes échographiques doivent conduire à une exploration chirurgicale des patientes à forte suspicion de torsion ovarienne afin de préserver le pronostic fonctionnel.

## Conflits d’intérêts

Les auteurs ne déclarent aucun conflit d'intérêts.

## References

[1] Anteby SO, Schenker JG, Polishuk WZ (1975). The value of laparoscopy in acute pelvic pain. Ann Surg.

[2] Hibbard LT (1985). Adnexal torsion. Am J Obstet Gynecol.

[3] Huchon C, Fauconnier A (2010). Adnexal torsion: a literature review. Eur J Obstet Gynecol Reprod Biol.

[4] Huchon C, Staraci S, Fauconnier A (2010). Adnexal torsion: a predictive score for pre-operative diagnosis. Hum Reprod.

[5] Shadinger LL, Andreotti RF, Kurian RL (2008). Preoperative sonographic and clinical characteristics as predictors of ovarian torsion. J Ultrasound Med Off J Am Inst Ultrasound Med.

[6] Nizar K, Deutsch M, Filmer S, Weizman B, Beloosesky R, Weiner Z (2009). Doppler studies of the ovarian venous blood flow in the diagnosis of adnexal torsion. J Clin Ultrasound JCU.

[7] Peña JE, Ufberg D, Cooney N, Denis AL (2000). Usefulness of Doppler sonography in the diagnosis of ovarian torsion. Fertil Steril.

[8] Hiller N, Appelbaum L, Simanovsky N, Lev-Sagi A, Aharoni D, Sella T (2007). CT Features of Adnexal Torsion. Am J Roentgenol.

[9] Bernhard LM, Klebba PK, Gray DL, Mutch DG (1999). Predictors of persistence of adnexal masses in pregnancy. Obstet Gynecol.

[10] Condous G, Khalid A, Okaro E, Bourne T (2004). Should we be examining the ovaries in pregnancy? prevalence and natural history of adnexal pathology detected at first-trimester sonography: Ovarian cysts in early pregnancy. Ultrasound Obstet Gynecol.

[11] Aggarwal P, Kehoe S (2011). Ovarian tumours in pregnancy: a literature review. Eur J Obstet Gynecol Reprod Biol.

[12] Tariel O, Huissoud C, Rudigoz RC, Dubernard G Presumed benign ovarian tumors during pregnancy. J Gynécologie Obstétrique Biol Reprod.

[13] Yen C-F, Lin S-L, Murk W, Wang C-J, Lee C-L, Soong Y-K (2009). Risk analysis of torsion and malignancy for adnexal masses during pregnancy. Fertil Steril.

[14] Galinier P, Carfagna L, Delsol M, Ballouhey Q, Lemasson F, Le Mandat A (2009). Ovarian torsion, management and ovarian prognosis: a report of 45 cases. J Pediatr Surg.

[15] Bider D, Mashiach S, Dulitzky M, Kokia E, Lipitz S, Ben-Rafael Z (1991). Clinical, surgical and pathologic findings of adnexal torsion in pregnant and nonpregnant women. Surg Gynecol Obstet.

